# A dual targeted β-defensin and exome sequencing approach to identify, validate and functionally characterise genes associated with bull fertility

**DOI:** 10.1038/s41598-017-12498-x

**Published:** 2017-09-25

**Authors:** Ronan Whiston, Emma K. Finlay, Matthew S. McCabe, Paul Cormican, Paul Flynn, Andrew Cromie, Peter J. Hansen, Alan Lyons, Sean Fair, Patrick Lonergan, Cliona O’ Farrelly, Kieran G. Meade

**Affiliations:** 1 Animal & Bioscience Research Department, Animal & Grassland Research and Innovation Centre, Teagasc, Grange, Co. Meath Ireland; 2 Weatherbys Scientific, Johnstown, Naas, Co Kildare Ireland; 3 Irish Cattle Breeding Federation, Bandon, Co. Cork Ireland; 4 Department of Animal Sciences, University of Florida, Gainesville, Florida USA; 5 Department of Biological Sciences, University of Limerick, Limerick, Ireland; 6 School of Agriculture and Food Science, University College Dublin, Belfield, Dublin 4 Ireland; 7 Trinity Biomedical Sciences Institute, Trinity College, Dublin 2, Ireland

## Abstract

Bovine fertility remains a critical issue underpinning the sustainability of the agricultural sector. Phenotypic records collected on >7,000 bulls used in artificial insemination (AI) were used to identify 160 reliable and divergently fertile bulls for a dual strategy of targeted sequencing (TS) of fertility-related β-defensin genes and whole exome sequencing (WES). A haplotype spanning multiple β-defensin genes and containing 94 SNPs was significantly associated with fertility and functional analysis confirmed that sperm from bulls possessing the haplotype showed significantly enhanced binding to oviductal epithelium. WES of all exons in the genome in 24 bulls of high and low fertility identified 484 additional SNPs significantly associated with fertility. After validation, the most significantly associated SNP was located in the *FOXJ3* gene, a transcription factor which regulates sperm function in mice. This study represents the first comprehensive characterisation of genetic variation in bovine β-defensin genes and functional analysis supports a role for β-defensins in regulating bull sperm function. This first application of WES in AI bulls with divergent fertility phenotypes has identified a novel role for the transcription factor *FOXJ3* in the regulation of bull fertility. Validated genetic variants associated with bull fertility could prove useful for improving reproductive outcomes in cattle.

## Introduction

While selection for production traits such as milk yield has been successful in dairy cattle there has been a simultaneous decrease in fertility^1^, the antagonistic relationship between the two traits ascribed to the evolutionary biology trade-off hypothesis^2,3^. Selection for increased male fertility is difficult as the heritability of fertility traits is much lower (2–4%) than that of production traits^4,5^ in cattle. Importantly however, genetic correlation between milk production and fertility is not in unity, and therefore, genetic improvement in both traits is possible with improved breeding selection^3^. Bull fertility and semen-related traits are important factors in maintaining overall herd fertility as semen from a single bull is generally used to breed large numbers of cows and heifers^6^ when used in artificial insemination (AI). While much research attention has focused on cow fertility traits, understanding of bull fertility traits is not as advanced^7^.

A successful outcome to a given service (i.e. birth of a live calf) is a combination of both male and female fertility^8^ and a significant proportion of reproductive failures in dairy cows has been attributed to bull sub-fertility^9^. However accurate measurement of bull fertility is complicated by different definitions of the phenotype and limited numbers of repeated measures per sire^10^. Sufficient numbers of repeatable bull fertility phenotypes are only currently available on bulls used in AI, where large numbers of inseminations can be used to reliably predict the fertility of an individual bull. Often these evaluations are undertaken by breeding companies which do not account for potentially confounding effects (e.g. herd, technician, cow genotype/parity), may not be publicly available^8^ or are complicated by the issue of compensable sperm traits^11^, where sperm numbers per insemination dose can be adjusted to improve fertility of sub-fertile bulls, thereby affecting fertility outcomes. *In vitro* assays (e.g. computer assisted sperm analysis of motility and morphology, flow cytometric measurement of sperm function and *in vitro* fertilisation) provide some quantitative data on sperm function but they explain only approximately 40% of the variation in bull field fertility^12^. Therefore, no single current diagnostic test can consistently and accurately predict fertility in bulls which produce morphologically normal semen^13^.

To overcome some of these limitations, multiple alternative approaches have been employed to identify genes regulating bull fertility; incorporating these markers into national selection indexes would lead to cumulative and permanent improvements in this critical trait. Both candidate gene and genome-wide approaches have been successfully used to understand the genomic architecture of bull fertility (as recently reviewed^7^). High levels of conservation of genes regulating some specific reproductive processes^14^ has enabled comparative genomic approaches to uncover novel candidate single nucleotide polymorphisms (SNPs) for bull fertility in a hypothesis driven manner^15^. Our earlier work uncovered a suite of β-defensin genes^16^, which are extensively expressed in the reproductive tract of the bull^17^ and on sperm^18^. While their functions have not been explored broadly in livestock species, knockout of a β-defensin gene cluster resulted in completely sterile male mice^19^. In humans, sequence variation in an orthologous gene to *BBD126* has been shown to contribute to significant subfertility in men^20^ and the peptide has been shown to mediate sperm binding to oviductal epithelium^21^. Therefore, we hypothesised that genetic variation in the bovine β-defensin genes may be associated with bull fertility.

The objective of the study was to identify genetic markers of male fertility in AI bulls whose sperm are morphologically normal but vary in field fertility. Two complementary approaches were undertaken using both targeted sequencing (TS) of 57 β-defensin genes and whole exome sequencing (WES) to identify, validate and functionally characterise genes associated with an adjusted animal model (AAM) of bull fertility. Use of TS allows the capture of sequence diversity in this newly discovered (and therefore largely unannotated) β-defensin gene family. WES is a novel method to capture and sequence all protein coding exons in the genome. Identified associated variants were then validated in an independent population of AI bulls and using sperm-binding functional tests.

## Results

### Selection of AI bulls with divergent fertility phenotypes

**Figure 1:**
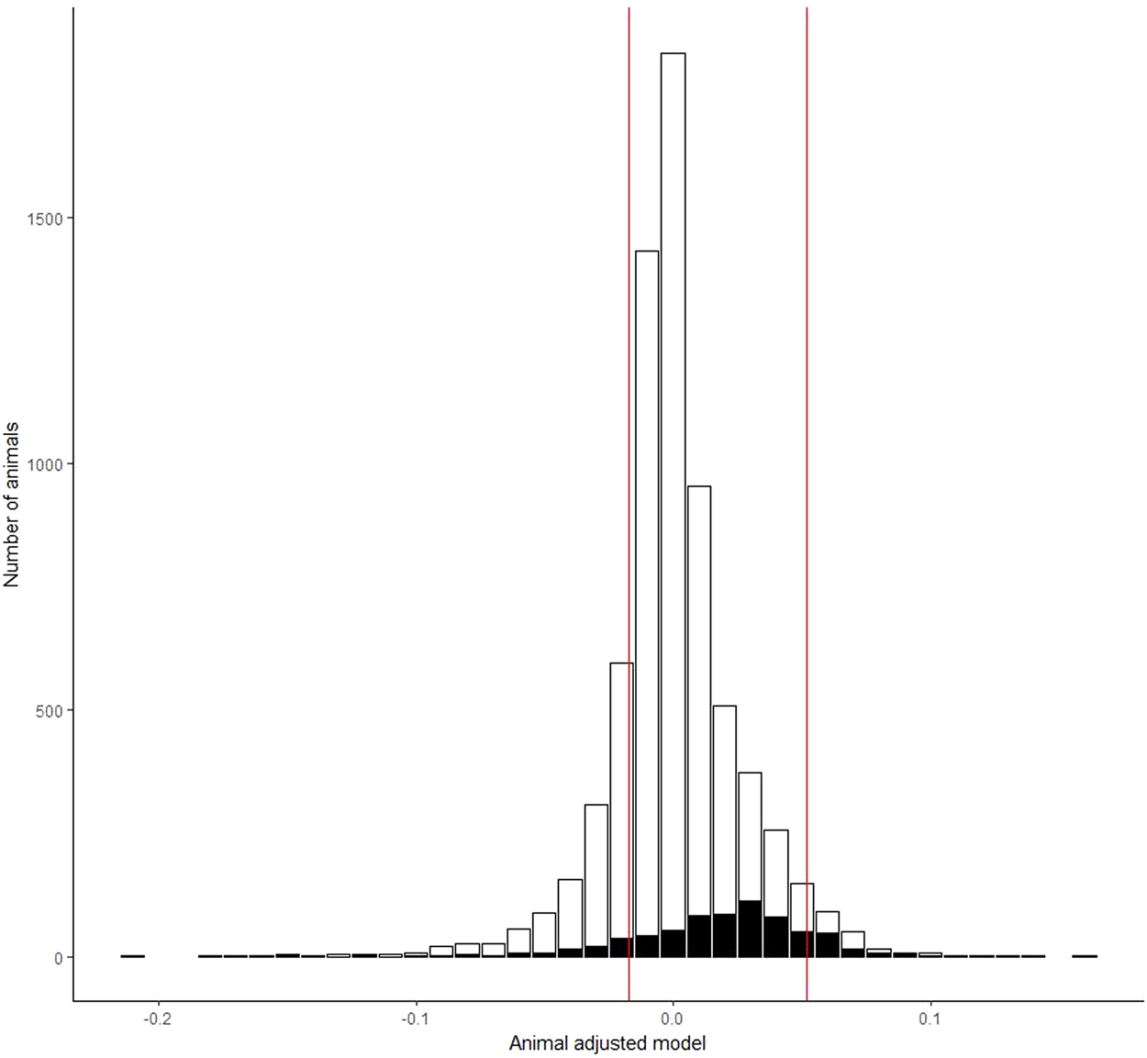
Range in fertility phenotype for all AI bulls assessed in this study. Adjusted animal model (AAM) bull fertility phenotypes calculated for all 7,000 sires (white) and 602 sires used for >1,000 inseminations with <25% being in 2013 (black). Bulls for sequencing were selected from the extremes of high and low fertility, defined as greater than one standard deviation from the mean, >0.052 or <−0.017, (outside region denoted by red lines). Restricting the samples to sires which have been used in >1,000 inseminations increased the reliability of the fertility phenotypes but removed sires identified as having very extreme pregnancy rates based on small numbers of inseminations.

The adjusted animal model (AAM) of fertility is a multiple regression mixed model of pregnancy rate adjusted for factors such as cow parity, herd, breed and time of insemination. AAM data for 7,000 bulls were filtered to the 602 bulls used in >1,000 matings. The phenotype ranged from −0.21 to + 0.12 with a mean of 0.0174. Extremes of fertility were identified as greater than one standard deviation from the mean, high- and low-fertility groups corresponded to an AAM range of <−0.017 or >0.052 (Fig. 1). Sires (n = 160) of mixed breed were selected for sequencing.

### Targeted β-defensin sequencing statistics

Baits were designed to target all 57 β-defensin genes and 1,000 bp upstream of each gene. A median of 88% of the 234,754 bp β-defensin targeted region had a sequencing coverage of >10X per sample and the median proportion with >30X coverage was 85% (Table S1). The total number of reads per sample ranged from 68,247 to 4,924,576 with a median value of 413,053. 9 of the 160 bulls were sequenced in duplicate in different captures and sequencing runs for quality control. After variant calling, the genotypes called in each duplicate were compared. One pair had only 64% identity and both were removed. The other 8 had an average genotype identity of 96.8% (95.9–97.4%). The copy with the highest call rate of each pair was retained for analysis.

### SNPs identified in the β-defensin gene cluster

**Figure 2:**
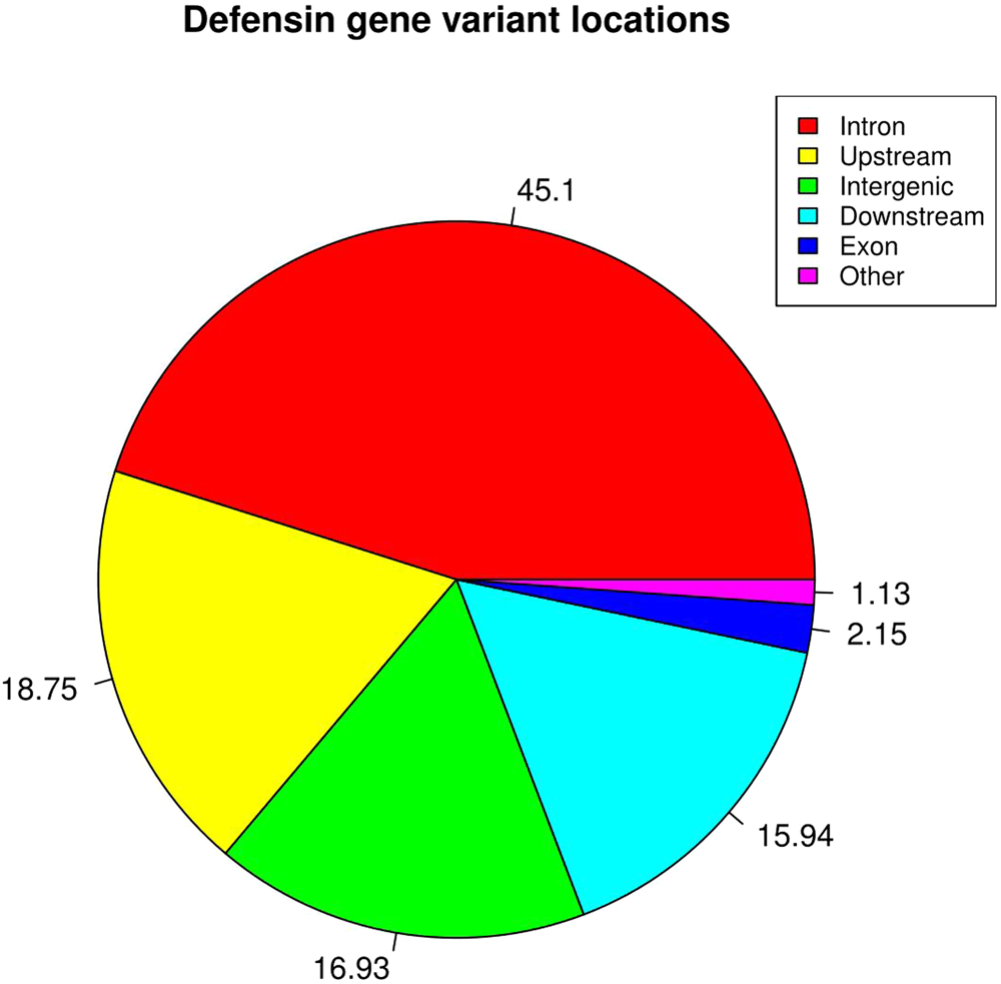
Genomic location of β-defensin genetic variants detected in bulls used in artificial insemination. Pie chart of percent of total SNPs identified in 160 bulls selected targeted sequencing of β-defensin genes. Genomic features where SNPs are located are identified in the key: intron (non-coding sections of DNA), upstream (5 k bp 5′UTR of a gene), intergenic region, downstream (5 k bp 3′UTR of a gene), exon (coding region), other (non-classified variants). Because the genes are found in four clusters a single SNP can have multiple annotations; for example, one may be downstream of one gene, upstream of a second and intergenic.

A total of 92,829 variants were identified from all TS reads. Of these, 3,134 remained after filtration and removal of all those with <80% call rate. This dataset consisted of 2,892 SNPs, 105 insertions and 137 deletions. A total of 45% of these variants were intronic, 18.7% were located upstream and 15.9% located downstream of the coding region and 16.9% were intergenic (Fig. 2). Only 2.15% of variants lay within exons. Fifty-nine SNPs, which caused nonsynonymous changes in the peptide sequences of 32 genes were identified. Two rare mutations changed the six-cysteine motif characteristic of β-defensins; rs477570826 causes a Cys34Ser change in the predicted gene ENSBTAG00000047421 (encoding *BD109*), removing the second cysteine and is heterozygous in two bulls. rs437613002 causes a Cys55Arg change in *BBD131*, removing the fifth cysteines, it was heterozygous in six bulls and one animal was homozygous for the alternate allele (data not shown).

### β-defensin gene association with bull fertility

**Figure 3:**
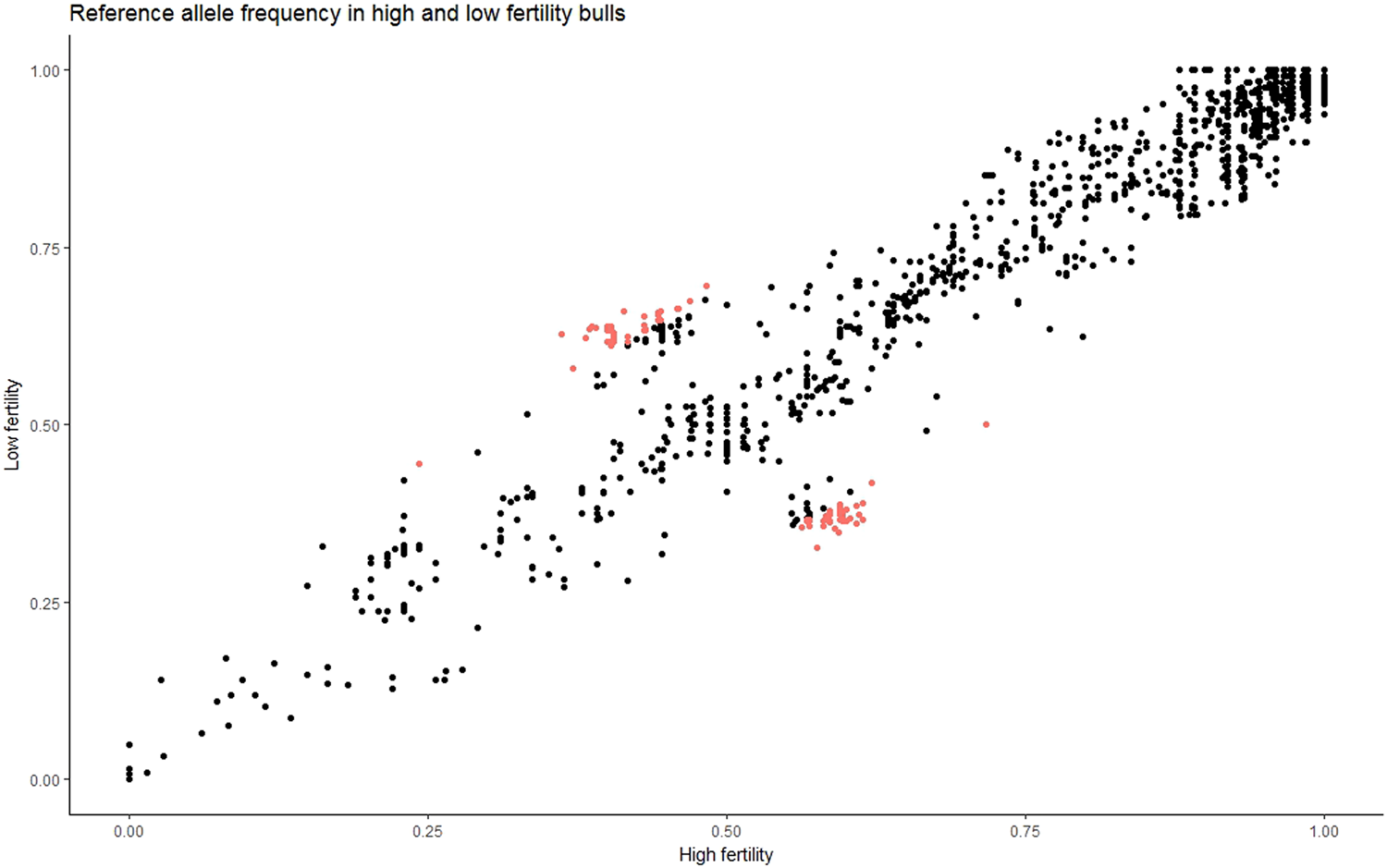
Allele frequencies of the SNPs identified in the targeted sequencing of β-defensin genes in bulls of high and low fertility. The frequency of the alternate allele in bulls of high fertility is shown on the x axis and the alternate allele frequency in bulls of low fertility on the y axis. SNPs which have a difference in SNP frequency of >20% between bulls of high and low fertility are highlighted in red. SNPs with a SNP frequency difference over 20% between groups are more likely to be under selection pressures, than SNPs with low SNP frequency differences.

**Figure 4:**
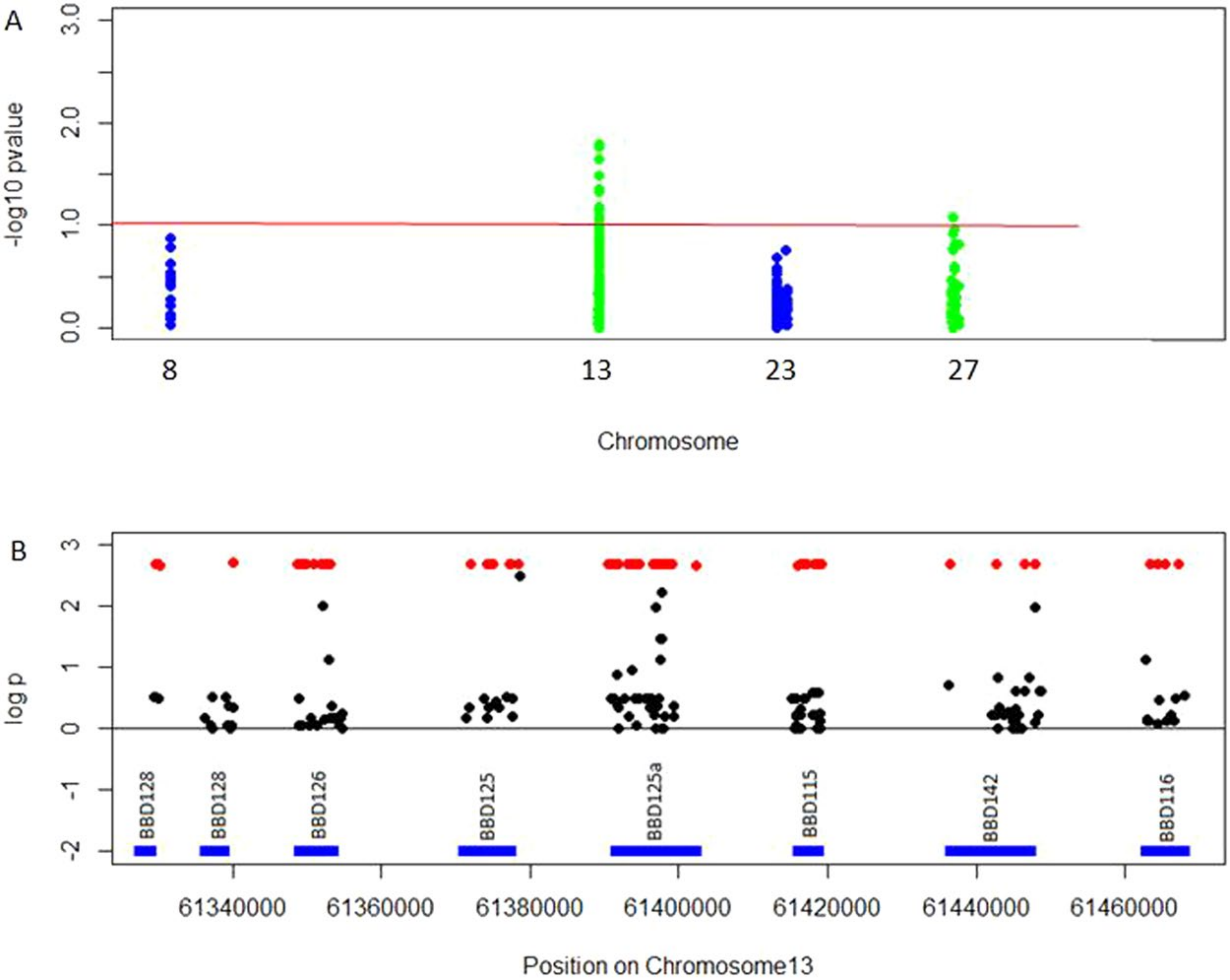
Significant association detected between β-defensin haplotype and bull fertility. (**A**) Association analysis of high quality SNPs and the adjusted animal model phenotype of fertility, -log_10_(*P*-value) is shown for the SNPs in the four β-defensin gene clusters on BTA8, BTA13, BTA23 and BTA27. The most significantly associated SNPs are all located on BTA13. Level of significance: unadjusted P-value < 0.01, indicated by red line. (**B**) –log_10_(*P*-value for all SNPs in the region on BTA13 containing the 98 SNPs found in a heterozygous haplotype in 9 sires (shown in red). The region contains 8 β-defensin genes, including *BBD126*.

Quality control (QC) removed 1,578 TS SNPs of very low minor allele frequency (MAF < 2.13%), 907 SNPs with <95% call rate, 913 SNPs not in Hardy-Weinberg equilibrium and 4 bulls with <95% call rate. The final TS data set comprised 1,399 SNPs in 149 bulls, with 25 on BTA8, 626 on BTA13, 356 on BTA23 and 392 on BTA27. The differential SNP frequencies in high and low fertility bulls are shown in Fig. 3. Following association analysis, the SNPs with the most significant association to bull fertility all lie on BTA13 (Fig. 4). One SNP (rs378043559) had a *P* = 0.00197 and 97 SNPs had the same *P-*value of *P* = 0.00202 (Table S2). Upon examination of the genotypes it became clear that all 98 SNPs were found as a haplotype, in 9 heterozygous sires (5 Holstein-Friesian, and 1 each of Limousin, Simmental, Charolais and Belgian Blue). All other bulls were homozygous for the reference allele. The SNP rs378043559 had a similar pattern of genotypes but also had one missing genotype call which changed the *P*-value. The 9 heterozygous sires were of average to high fertility (0 to 0.07, mean 0.04, sd. 0.027). The 98 SNPs were located between positions 61,329,700 and 61,467,209, a region of 137.5 kb containing the genes *BBD128*, *BBD127*, *BBD126*, *BBD125*, *BBD115*, *BBD142* and *BBD116*. Of the 98 SNPs in the haplotype, 3 were located within coding regions, a nonsynonymous SNP in *BBD115* (Ser52Asn) and two synonymous SNPs in *BBD126* and *BBD125*. 76 SNPs were located within introns, 5 were located downstream and 13 located upstream of genes and 18 were intergenic.

### Exome sequencing statistics

In the 24 bulls in which all exons were sequenced, coverage of on-target regions (regions of the exome targeted by our sequence capture probes) was 98.6%. A mean of 39 million reads per sample were retained following read filtering, ranging from 23,716,853 to 78,048,261. The proportion of reads uniquely aligned to the genome averaged 94%, with 3.45% PCR duplication of reads. Percentage exome coverage was 95.5% at a depth of 2X, and 69% at 10X depth, ranging from 95% to 98% and 49% to 88% respectively. At higher depth, the percentage of the targeted genome sequence with reads mapping was low with a mean of 33% at 20X and 12% at 30X, ranging from 11% to 69% and 2% to 50% respectively. The 69% of the genome sequenced at 10X coverage was deemed sufficient for SNP discovery, as shown previously^22^. Full details of sequencing statistics are given in Table S3.

### WES variant identification and association analysis with bull fertility

**Figure 5:**
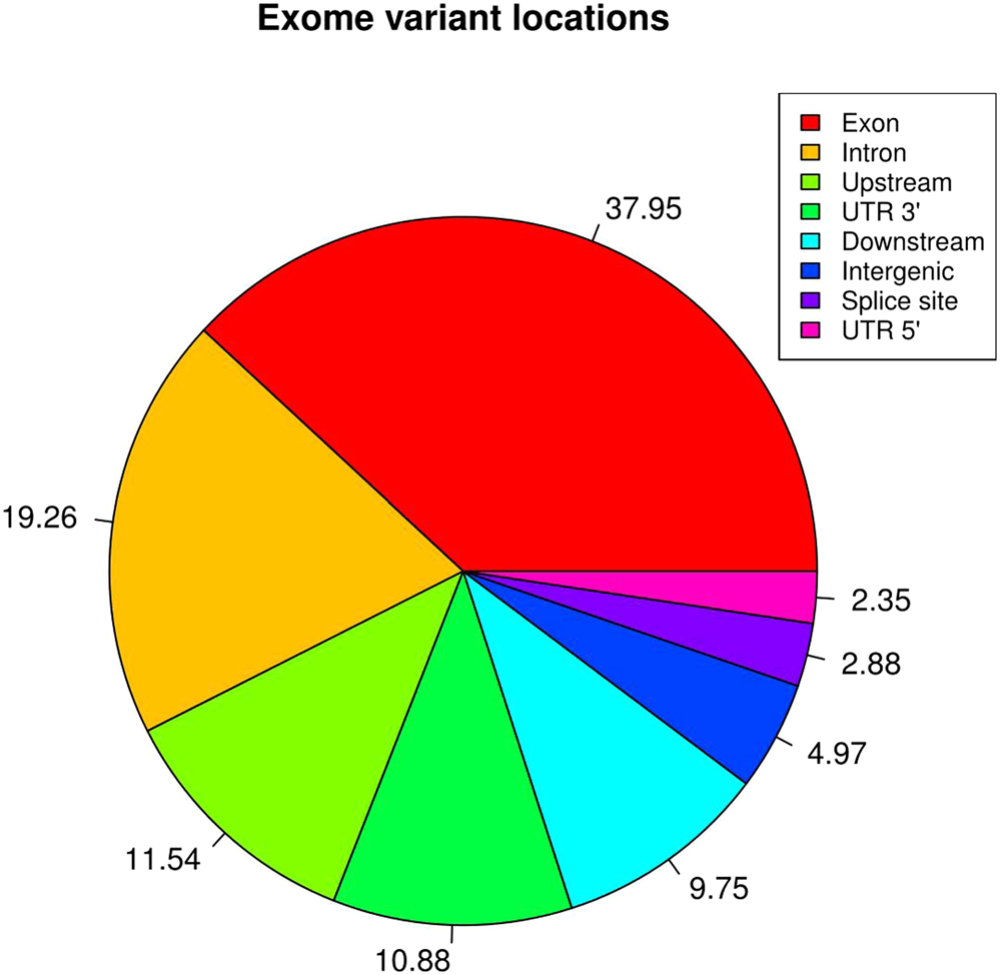
Genomic location of genetic variants detected in bulls used in artificial insemination using WES. Pie chart of percent of total SNPs identified in 24 bulls selected for whole-exome sequencing located in various genomic features. Genomic features where SNPs are located are identified in the key: Exon (coding region of DNA), intron (non-coding region of DNA), upstream (non-coding region 5 k bases 5′ of a gene), UTR 3′ (untranslated region 5 k bases 3′ of a gene), downstream (3′UTR of a gene), intergenic region, splice site (exon boundaries), UTR5′ (Untranslated region containing transcription factors).

**Figure 6:**
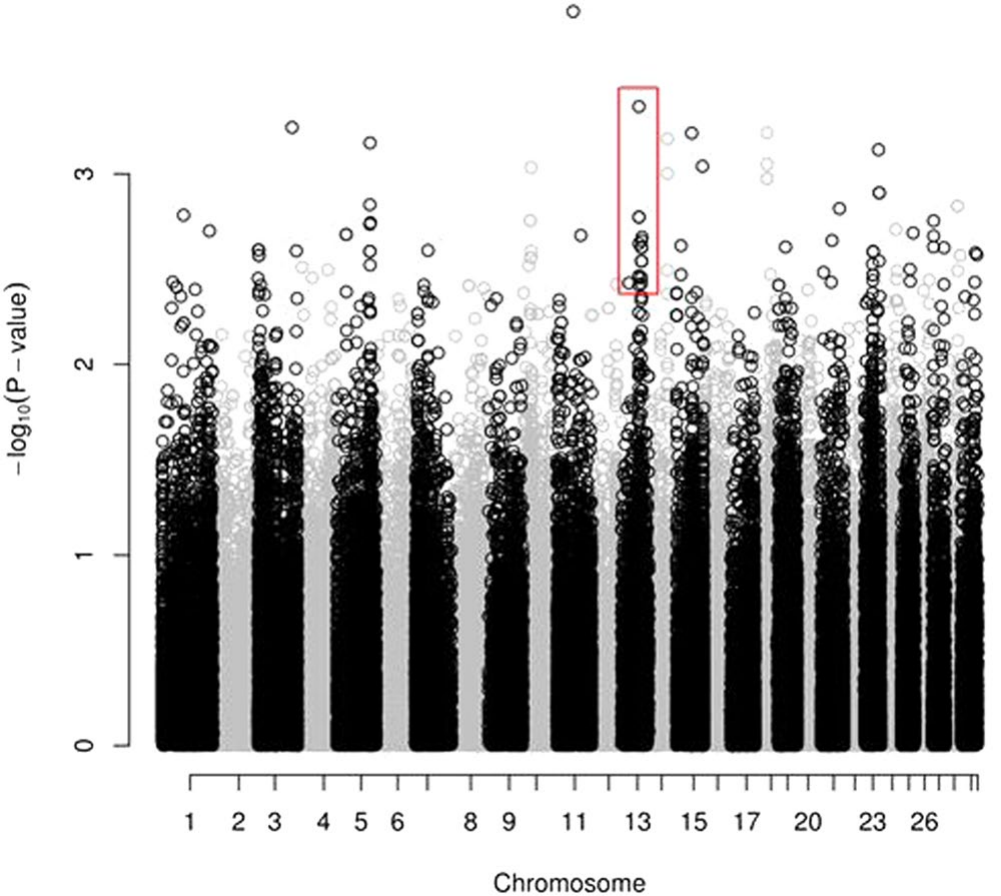
SNP Association of genotypes and fertility for whole-exome sequence data. Manhattan plot shows the association of variants identified by whole-exome sequencing and their associated *P*-value with the adjusted animal model fertility phenotype. The *P*-value for each variant association is on the y-axis. The chromosomal position of each variant is on the x-axis. In total, there are 144,178 variants after quality control shown. Red box indicates cluster of associated SNPs with fertility located on chromosome 13, located near the β-defensin gene cluster (16 out of 38 SNPs in this region are within the β-defensin gene region).

A total of 3,437,419 variants were identified in all 24 divergent fertility bulls. Removal of variants outside the targeted regions with a read coverage threshold of <5X, reduced this number to 289,210. Of these 12,124 were insertions and 13,048 were deletions, leaving 264,038 SNPs. Further quality control using GenABEL removed SNPs with an overall call rate of <80% or minor allele frequency of <5% and samples with a call rate of <90% of SNPs, leaving 144,178 variants. Following association analysis, 484 variants had an association with the bull fertility at an unadjusted significance level of *P* < 0.01. A list of all significantly associated variants is shown in Table S4 and the genomic location of genetic variants detected is shown in Fig. 5. As shown in the Manhattan plot in Fig. 6, clusters of SNPs associated with fertility are found on chromosomes 3, 13 and 23. 38 SNPs were located on chromosome 13 and associated with fertility (unadj. P-value < 0.01). Of these, 16 (highlighted in red) were located within positions 61,000,000 and 62,000,000. Eleven of the top 20 most associated SNPs were predicted to be in the intron region of a gene, 7 were predicted to be located in exons (4 synonymous, 1 missense and 2 unknown), 2 were predicted to be non-coding, and 2 were upstream of their respective genes (Table S4). The most significantly associated SNP was in the intron region of a predicted gene on BTA11 at position 49,866,493 with an unadj. *P* = 0.00014. It is predicted to be in the U6 novel snRNA in bulls, a pseudogene of the small non-coding RNA class.

A SNP in the signal regulatory protein alpha, SIRPA, gene located on BTA13 at position 53691410 was the second most significantly associated variant (*P* = 0.00044). The third most significantly associated SNP, rs109065788, is synonymous and located on BTA3 in the exonic region of the forkhead box J3 (*FOXJ3*) gene.

### Gene ontological analysis of WES variants

A list of genes which contain or are nearest to the 484 SNPs identified via WES and associated with fertility was used for Gene Ontology (GO) analysis. Approximately 220 genes were included in gene ontology over-representation analysis, as the DAVID database did not contain all submitted Ensembl gene IDs. Two functional categories were significantly over-represented in this dataset after Benjamini-Hochberg correction for multiple testing; ‘glycoprotein’ (*P* = 0.0056) and ‘glycosylation site: N-linked’ (*P* = 0.00024). There are 27 genes involved in ‘glycosylation site: N-linked’ and 28 genes involved in ‘glycoprotein’. Of the 12 over-represented terms and keywords identified, 8 contained innate immunity terms, although ‘disulphide bond’ and ‘glycoprotein’ have also been linked to the innate immune system, specifically to β-defensins. A full list of GO terms over-represented in the whole-exome sequencing dataset is shown in Table S5.

### Validation of SNP associations in independent population of AI bulls

**Figure 7:**
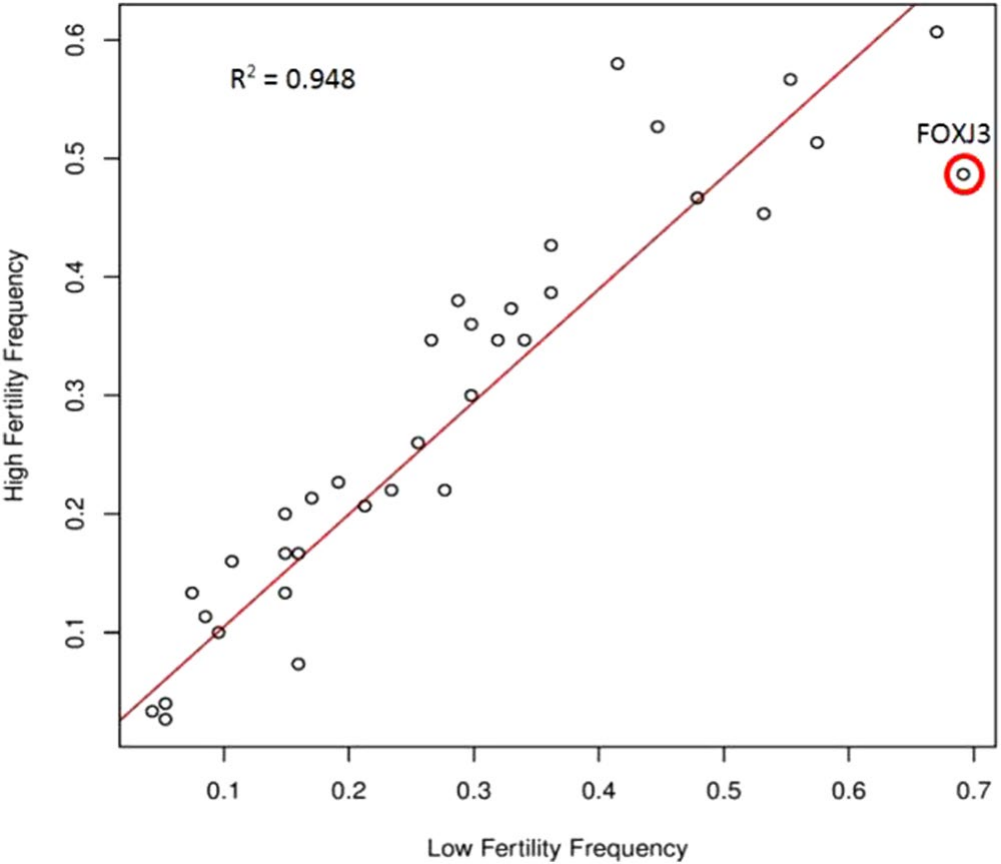
Validated SNP frequencies in independent population of AI bulls. Scatterplot of SNP frequencies of validated SNPs (n = 42) with x-axis = low-fertility bulls SNP frequencies and y-axis = high-fertility bulls SNP frequencies (n = 123 bulls). Red line = line of regression. R^2^ = Pearson’s correlation coefficient. The most significantly associated SNP in the validation dataset (located in FOXJ3) is highlighted in red (SNP frequency difference = 21% (48–69%)).

A SNP genotyping assay for 58 SNPs identified as being associated with bull fertility from TS and WES datasets, of which 42 passed all QC filters and were validated in an independent population of 123 bulls. Allele frequencies of validation SNPs along the line of regression, with an R^2^ coefficient of determination equal to 0.948, are shown in Fig. 7. The SNP most associated with bull fertility in the validation dataset, rs109065788, is in the exonic region of *FOXJ3* (adj. P = 0.069) (Table 1). *FOXJ3* is located on BTA3 at position 104,587,541 and results in a synonymous mutation. The minor allele of the SNP was found at a frequency of 69% in the low fertility bulls and 48% in high-fertility bulls. The second most associated SNP in this dataset was in an unannotated gene (*LOC536148*) on BTA30 which is orthologous to A-kinase anchor protein 17B in mice and humans.

**Table 1:** Validated variants associated with adjusted animal model fertility phenotype.

Gene	CHR	SNP	Location	Number genotyped	BETA	SE	R^2^	T	Frequency ‘low’	Frequency ‘high’	*P-*value	BH
*FOXJ3*	3	rs109065788	104587541	101	0.01473	0.0045	0.095	3.227	0.691	0.486	0.0016	0.069
*LOC536148*	30	rs42198966	3481991	110	-0.007747	0.0029	0.059	-2.625	0.414	0.58	0.0099	0.203
*LOC100337009*	27	rs437191942	6225144	110	0.01466	0.0063	0.047	2.324	0.085	0.113	0.0220	0.300
*KIAA1549*	4	rs378655691	103370232	106	-0.01466	0.0068	0.041	-2.131	0.074	0.133	0.0354	0.363
*BBD124*	13	rs43710895	61615527	106	0.007818	0.0042	0.031	1.85	0.297	0.36	0.0671	0.479
*BBD123*	13	rs43710844	61595572	102	0.007353	0.0040	0.031	1.795	0.531	0.453	0.0756	0.479
*SIRPA*	13	10509	53691410	108	0.01016	0.0057	0.028	1.757	0.095	0.1	0.0818	0.479

Table of validated variants associated with the adjusted animal model fertility phenotype *P*-value < 0.1. Gene = SNP annotated to nearest gene; CHR = Chromosome number; SNP = SNP identifier; Location = Chromosomal position (base-pair); Number genotyped = Number of bulls genotyped; BETA = Regression coefficient; SE = Standard error; R2 = Regression r-squared; T = Wald test (based on t-distribution); Frequency ‘low’ = SNP frequency in low-fertility bulls; Frequency ‘high’ = SNP frequency in high-fertility bulls; P = Wald test asymptotic *P*-value; BH = Benjamini-Hochberg multiple testing correction.

### Functional assessment of haplotype on sperm binding to oviductal epithelium

**Figure 8:**
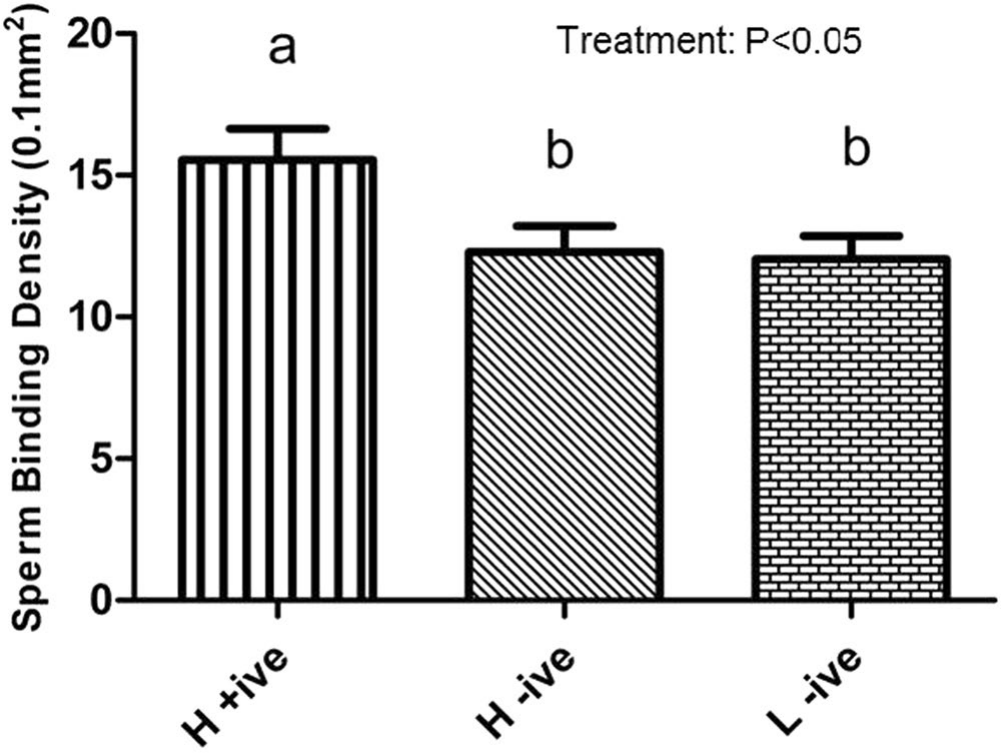
Significant effect of β-defensin haplotype on sperm binding to oviductal epithelium. Binding density of sperm from bulls of varying β-defensin haplotype (high fertility with β-defensin haplotype, H + ive; High fertility without β-defensin haplotype, H-ive; and low fertility without β-defensin haplotype, L − ive) to bovine oviductal epithelial cell explants. n = 4 biological and a further 3 technical replicates per group. Twelve straws per haplotype were assessed. Vertical error bars represent s.e.m. Different superscripts refer to statistically significant differences (P < 0.05).

The haplotype spanning several β defensin genes identified through TS was shown to have a significant effect on sperm binding density to bovine oviductal epithelial cell (BOEC) explants (P < 0.05; Fig. 8). Sperm from bulls carrying the β-defensin haplotype (H + ive) bound to BOEC explants at a greater density than sperm from bulls without the haplotype. The haplotype is found in bulls of high fertility, therefore, the binding of sperm from bulls possessing the haplotype (H + ive) was compared to both bulls of equivalent high fertility but with the reference allele at all haplotype snps (H − ive) and bulls of low fertility with the reference allele at all haplotype SNPs (L − ive) to ensure differences in binding did not reflect unrelated differences in fertility. Sperm binding density was 15.5 ± 1.11, 12.3 ± 0.91, 12.0 ± 0.83 (mean ± s.e.m) for sperm from H + ive, H-ive, and L-ive bulls, respectively. There was a significant effect of bull on binding to BOEC explants (*P* < 0.05) but there was no bull x haplotype interaction detected (*P* > 0.05).

## Discussion

Dairy enterprises are the highest users of AI bulls for breeding, and as these bulls have their semen intensely pre-screened by breeding companies they tend to represent not only elite genetics but also higher than average fertility. Despite this, pregnancy rates can still fall as low as 25%^23^. Given their important role in reproductive success, variation in male traits has been poorly investigated^24^ and reliable predictive fertility-related biomarkers are urgently required^25^.

One mechanism to enhance bull fertility is to identify genes regulating this critical trait to enhance the accuracy of artificial selection and breeding. Therefore, in this study, dual TS and WES of bulls with divergent fertility was performed using a comprehensively defined model of bull fertility. This model was developed following a large-scale analysis of records from >350,000 artificial inseminations across almost 3,000 herds to define a pertinent male fertility trait for a seasonal production system.

In the current study, custom-designed probes were used to capture and sequence all 57 currently known β-defensin genes as well as the estimated 220,000 exons annotated in the current draft of the bovine genome. These complementary approaches were chosen as our earlier work identified a panel of novel β-defensin genes, highly expressed in the reproductive tract of the bull^17^, which have not all been annotated in the most recent draft of the bovine genome. A total of 2,836 SNPs across the 4 clusters of β-defensin genes were identified, 37% of which are novel and have not been previously described in cattle.

Association analysis identified a β-defensin haplotype spanning seven genes on BTA13 associated with bull fertility (*BBD128*, *BBD127*, *BBD126*, *BBD125*, *BBD115*, *BBD142* and *BBD116*). SNPs in this region may explain previously published genome-wide associations between this region and bull fertility^7^. At least 2 of the 4 β-defensin clusters are copy number variable^26,27^ and therefore these regions were difficult to sequence and assemble unambiguously. It is possible therefore that these genes, and the novel SNPs identified herein explain a higher proportion of phenotypic variation in bull fertility that can currently be explained using current genome-wide association approaches.

As a new technological approach, WES has been employed in a few previous studies, with one recent application to successfully uncover causative genetic variants underlying defective embryo development in Holstein and Brown Swiss cattle breeds^28^. In total, WES identified 144,178 variants in bulls with divergent fertility, 484 of which were associated with fertility (unadj. P < 0.01). After validation in an independent population, a SNP in U6, a snRNA gene/pseudogene remained significantly associated. Although known to be highly-expressed in the testes of bulls^29^, little functional information on this gene exists, and therefore future studies are required to verify its function, especially in relation to fertility. In addition, another associated SNP variant in the signal-regulatory protein alpha (*SIRPA*) gene was identified. *SIRPA* is a promising candidate gene for spermatogenic impairment and male fertility^30^. Another associated synonymous variant was in the exonic region of the forkhead box J3 gene (*FOXJ3*). Furthermore, gene ontology analysis identified the terms “glycoprotein’ (*P* = 0.0056) and ‘glycosylation site: N-linked’ (*P* = 0.00024) as significantly over-represented terms in the SNP dataset, reaffirming the emerging important relationship between the immune system and male fertility^31^.

To validate the genetic variants identified via both TS and WES approaches, validation was performed in an independent dataset of AI bulls and one SNP validated as significantly associated with bull fertility (adj. P < 0.1). The SNP most associated with the AAM fertility phenotype was in the *FOXJ3* gene and confirmed the finding from the WES analysis, with a SNP frequency differential between low and high-fertility bulls of 21% (48–69%), Fig. 7. *FOXJ3* is a transcription factor, and a recent publication showed that this gene is required for the survival of spermatogonia in mice^32^. This bovine SNP may be linked to another causal variant or alternatively, it has been shown in humans that synonymous mutations can affect mRNA abundance and protein concentrations, and can alter substrate specificity via altered protein conformations^33^. The second most associated SNP in the validation dataset was located in an unannotated gene (*LOC536148*) (adj. P = 0.203) on BTA30 which is orthologous to A-kinase anchor protein 17B in mice and humans, and is thought to play a structural role in the cytoskeletal scaffold of sperm^34^. Further studies are now warranted to determine the function of these genes in bovine and identify potential causal variants linked to bull fertility.

A missense SNP in the β-defensin gene *BBD123*, and an upstream variant in *BBD124* were identified as significantly associated with bull fertility through WES but they did not survive correction for multiple testing in the validation population (unadj. *P* = 0.0671 and 0.0756, respectively). These genes are both located in the 19 β-defensin genes on *BTA13* reported by our group to be expressed in the reproductive tract of the bull^35^. As putative fertility related genes in cattle were previously successfully identified on the basis of cross-species conservation^15^, in conjunction with a documented role for β-defensin in mediatinjg sperm-binding^21^, a sperm-binding assay was used to investigate the functional importance of the significantly associated β-defensin haplotype uncovered in this study. The β-defensin haplotype was only found in high fertility bulls and sperm from these bulls showed significantly higher binding to oviductal epithelium than similar bulls without the associated haplotype. Thus, sperm from these bulls appear to have an enhanced capacity to establish an oviductal sperm reservoir in preparation for ovulation and this may improve the chances of successful fertilisation. Currently, the identity of binding molecule(s) for BBD126 on epithelial cells is unknown^36^; however, the expression pattern of BBD126 on the bull sperm head^37^ appears to be consistent with the location and pattern of sperm attachment to the oviductal epithelium. Fertility is a complex multifactorial phenotype and these results confirm a contributory role for β-defensins in mediating sperm function in cattle. Moreover, this haplotype spans the region containing *BBD126*, which we have recently shown to promote sperm motility in cattle^38^.

## Conclusion

Given the important role of the male in determining herd fertility in cattle and the pace of genetic gain now possible with the advent of genomic selection, multiple approaches are required to unravel the complexity of bull fertility. Despite the small effective population size in most cattle breeds^39^, and the highly selected nature of AI bulls, our results show that novel fertility-associated SNPs are polymorphic and therefore amenable to selection. This study represents the first comprehensive catalogue of genetic variation in bovine β-defensin genes and the first whole-exome sequencing of divergent fertility AI bulls. This dual approach has successfully identified novel variants in both β-defensin and *FOXJ3* genes potentially regulating reproductive function, and these biomarkers may contribute to future strategies to breed for improved bull fertility.

## Methods

### Phenotypic analysis and sample selection

The Irish Cattle Breeding Federation (ICBF) collated male fertility data for 7,000 bulls used in AI in Ireland over four years (2010–2013 inclusive). Data from all recorded artificial inseminations were provided by ICBF and used to calculate bull fertility based on an adjusted animal model (AAM). AAM is a multiple regression mixed model of pregnancy rate, where a cow/heifer was confirmed to be pregnant to a given service either by a calving event and/or whereby a repeat service (or a pregnancy scan) deemed the animal not to be pregnant. Females which were culled or died on farm were omitted. The model was then adjusted for various factors, including semen type (frozen, fresh), cow genotype, parity of cow, month of service, day of the week when inseminated, service number, herd, AI technician, bull breed. Finally, the estimate from the model was weighted for number of insemination records. This gave the AMM which was expressed relative to the mean of the population for all bulls^8^. Of these, only bulls which had >1000 inseminations were selected, and furthermore as bulls were selected in 2013, inseminations from that year had not yet produced live calves and therefore bulls with >25% of their straws used in 2013 were removed, reducing the total number of bulls suitable to 602. Bulls used in AI have already undergone extensive pre-screening and have passed all the pre- and post-freezing semen quality control checks in the AI centre prior to release into the field. Infertile bulls which got no/few females pregnant were either screened out as part of the quality control checks or were not used extensively in AI as the bulls were retracted from use once extremely low fertility was captured by AI companies; therefore, these bulls were not part of the dataset as they did not reach the threshold of >1000 inseminations. Extremes of high and low fertility were defined as greater than one standard deviation from the mean, 160 bulls were chosen for final TS, consisting of 82 Holstein-Friesian, 21 Belgian Blue, 14 Limousin, 11 Aberdeen Angus, 10 Charolais, 6 Simmental, 5 Hereford, 5 Jersey, 2 Montbeliarde, 2 Norwegian Red and 2 Shorthorn. For WES, a subset of 24 bulls which had been used for TS were selected. High-fertility Holstein-Friesian ranged in between 0.05–0.07 for AAM, and the low fertility bulls ranged between −0.12 and −0.03 (n = 6/group). High-fertility Limousin ranged between 0.06–0.07, and low-fertility bulls ranged between −0.09 and −0.06 (n = 3/group). Belgian blue high-fertility bulls ranged between 0.05 and 0.08 with low fertility between −0.04 and −0.02 AAM (n = 3/group). Pedigree analysis was performed to minimise the selection of related individuals (pedigree score of relatedness <0.2).

### Targeted sequencing analysis

#### Library preparation

DNA was extracted using the Maxwell® 16, per manufacturer’s instructions. Genomic DNA (gDNA) was purified using Zymo Research’s DNA Clean and concentrator ™ kit (Zymo Research, Irvine, Ca, USA) and heated to 52 °C for 2 min prior to concentration estimation. gDNA concentrations were estimated using the Qubit® dsDNA BR Assay Kit for use with the Qubit® 2.0 Fluorometer. A total of 200 ng DNA was sheared with a Bioruptor Plus sonicator (www.diagenode.com) and the fragment length of approximately 600 bp confirmed using a Bioanalyzer (Agilent, California). End-repair, A-tail and adapter ligation were carried out as specified in the TruSeq Nano DNA LT Sample Prep protocol (Illumina Ltd.). Twenty-four samples were chosen at random to barcode and pool for sequence capture.

#### DNA capture and sequencing

The complete genomic sequences for all known β-defensin genes plus 1000 bp upstream of the transcription start site (a total of 387 kb) were used for Roche Nimblegen SeqCap EZ Developer bait design, per standard protocols. Where possible, baits which align to only one genomic location were used. This criterion was relaxed to allow baits which align up to five locations for important exonic regions. In total, it was possible to design baits to target 235 kb; the unsuccessfully targeted regions consisted primarily of intronic and intergenic repetitive sequences. The suggested dual-capture protocol was used. Following pre-capture amplification, 24 libraries were pooled to form 1 μg of DNA which was hybridised with oligos and baits overnight, captured, amplified, hybridised with another aliquot of the baits overnight, amplified and cleaned. Libraries were quantified using Qubit Hi Res (Life Technologies) and run on an Illumina MiSeq at 10pM with 1% PhiX (300 bp paired-end protocol).

#### Variant discovery

Reads were assigned to appropriate samples using the barcode sequence. Paired end reads were quality filtered and adaptor trimmed. Reads were aligned to the *Bos taurus* genome UMD3.1. Picard tools were used to sort and order the files. Enrichment, insert size and alignment metrics were calculated using the Genome Analysis Toolkit (GATK). Variant discovery was also performed using GATK, following the Best Practice Pipeline (https://www.broadinstitute.org/gatk/); variants were called individually using the Haplotype Caller and joint genotyping performed on all samples simultaneously using GenotypeGVCFs. The results were filtered using GATK Variant Filtration with the following parameters: filter out variant calls if located within a cluster where three or more calls were made in a 10 bp window [clusterWindowSize 10]; filter out variant if there were at least four alignments with a mapping quality of zero (MQ0) and if the proportion of alignments mapping ambiguously corresponds to 1/10^th^ of all alignments [MQ0 > = 4 && ((MQ0/(1.0 * DP)) > 0.1)], DP: total (unfiltered) depth over all samples; filter out variants which were covered by less than 5 reads [DP < 5]; filter out variants having a low quality score [Q < 50]; filter out variants with low variant confidence over unfiltered depth of non-reference samples (QD) [QD < 1.5]; filter out variants based on strand bias using Fisher’s exact test: FS > 60.0 for SNP calling, FS > 200.0 for indel calling. In house Perl scripts were also written to identify any individual genotypes identified as heterozygous with an allele ratio of >80:20 and any SNP which had a read depth of less than 8 in a given individual was coded as missing data for that individual.

#### Association analysis

Association analysis was performed using the R package GenABEL^40^. Quality control was performed with the check.marker function and SNPs were examined for association to fertility using breed and the number of inseminations performed as fixed effects.

### Whole exome sequencing

#### DNA extraction and purification

Genomic DNA for 24 selected bulls was extracted using the Maxwell® 16, per manufacturer’s instructions. Genomic DNA (gDNA) was purified using Zymo Research’s DNA Clean and Concentrator ™ kit and heated to 52 °C for 2 min prior to concentration estimation. gDNA concentrations were estimated using the Qubit® dsDNA BR Assay Kit for use with the Qubit® 2.0 Fluorometer.

#### Probe design

Roche Nimblegen, SeqCap EZ Developer Library was purchased from Roche (Roche NimbleGen, WA, USA) to perform a custom-designed sequencing of the bovine exome. Liquid capture probes were designed to target all exons annotated in the *Bos taurus* UMD3.1 genome, plus 100 base pairs of 5′UTR, per standard protocols. An in-house Perl script was used to identify target regions. Probes were manufactured by Roche (Roche NimbleGen). Magnetically labelled, oligonucleotide probes capture the target regions of DNA during library preparation. One hundred base pairs of 5′ untranslated region (UTR) were also included for all genes. In total, there were 227,647 exons in the genome transfer file. Of these, 202,899 were targeted in this design, covering 56,671,697 bp. Less than five total matches and five mismatches were allowed. Of these probes, 92.5% were unique to a single genomic position, and 4.5% had only 2 possible matches within the genome. The length of probes was 200 bp with approximately 2.1 million probes made to cover the exome. Probes were also designed to target mitochondrial DNA and were 1/5^th^ of the concentration of nuclear DNA. Using probes unique within the genome, that is probes that map exactly once in the genome, covers only 80% of the target regions; whereas using probes that map up to 5 times in the genome covers approximately 98.4% of the target regions.

#### Library preparation

Library preparation for WES was commercially performed (Clinical Genomics, Canada) following the Roche Nimblegen SeqCap EZ Developer Library protocol, as per the manufacturer’s instructions. Briefly, 100 ng gDNA of each sample were prepared using the TruSeq Nano DNA Library Prep Kit. This step involved shearing of gDNA using Bioruptor DNA shearing and Agencourt AMPure XP beads, end-repair of fragments, adapter ligation, LM-PCR and quality control. The strategy to hybridise fragments of gDNA to the exome baits was to make one equimolar pool of all 24 gDNA libraries. This pool was then split into four separate pools and hybridisation captures were performed on each pool with oligonucleotide probe capture reagents, resulting in 1X capture for every 6 samples. All captures were then re-pooled and split into four groups for sequencing on four HiSeq. 2500 lanes. Pooling ensures that samples were mixed prior to capture, and to give an even distribution of sample to each sequencing lane.

#### Data analysis

Sequencing files (FASTQ) were assessed for quality using FastQC. FASTQ files were trimmed using Trim Galore! in order to remove contaminant adapter sequences. A Phred quality score threshold of 25 was applied, to discard poor quality base calls, and to reduce the effect of incorrect base calling. All remaining reads were aligned to the *Bos taurus* UMD3.1.70 genome using the Burrows-Wheeler Aligner (BWA) ‘sampe’ algorithm with default parameters^41^. Picard Tools was used to convert the resulting SAM file to BAM format, sort and index BAM files, and to remove PCR duplicates from all BAM files. Alignment summary metrics, insert size metrics, and PCR duplicate metrics were all collected. GATK’s Depth Of Coverage walker determined coverage levels per interval.

#### Variant calling

Variant calling and genotyping across all 24 animals was performed using Genome Analysis Toolkit (GATK), following the GATK best practice guidelines for WES^42^. Local re-alignment around indels was performed using the GATK tools: Realigner Target Creator, Indel Realigner and Fix Mate Information. Base quality score re-calibration via GATK Base Recalibrator was then applied, which recalibrates scores around known variants. Haplotype Caller walker was used to call mutations on BAM files with Phred-scaled emit and call confidences of 30, in ‘GVCF’ mode and with a BED file of the exome targets. This BED target file is used by the walker to identify regions in the genome which were active/variable, which were marked for local de-novo assembly of reads aligning to such regions.

#### SNP filtering

Strict hard-filtering of variants was performed to remove variants of low quality and which fall outside certain parameters, to reduce the number of false positives. The parameters for hard filtering of variants were as follows: Filter out variant calls if located within a cluster where three or more calls were made in a 10 bp window [clusterWindowSize 10]; filter out variant if there were at least four alignments with a mapping quality of zero (MQ0) and if the proportion of alignments mapping ambiguously corresponds to 1/10th of all alignments [MQ0 > = 4 && ((MQ0/(1.0 * DP)) > 0.1)], DP: total (unfiltered) depth over all samples; filter out variants which were covered by less than 5 reads [DP < 5]; filter out variants having a low quality score [Q < 50]; filter out variants with low variant confidence over unfiltered depth of non-reference samples (QD) [QD < 1.5]; filter out variants based on strand bias using Fisher’s exact test: FS > 60.0 for SNP calling, FS > 200.0 for InDel calling, similar to other SNP filtering protocols^43^. In house Perl scripts were also written to identify any individual genotypes identified as heterozygous with an allele ratio of >80:20 and any SNP which had a read depth of less than 8 in an individual and code them as missing data.

#### SNP association

The R package GenABEL^40^ was used to perform association analysis between fertility and SNP genotypes. A custom R file converted a Variant Call Format file containing all variants from GATK Haplotype Caller to GenABEL format. A linear mixed model approach analysed each SNP separately for association with a phenotype, which allows for fixed and random effects. SNPs identified were analysed as continuous variables, in which case an allelic effect will be estimated. The null hypothesis is that there was no association between the SNP and the fertility trait. This model assumed a linear relationship between the trait and genotype as well as a common variance at each genotype. SNPs associated with the AAM fertility phenotype (p < 0.01) were sorted based on their unadj. *P*-value.

### Accession Codes

This data set was submitted to NCBI Gene Expression Omnibus under accession number GSE41637 and is available in ArrayExpress as E-GEOD-41637.

## Validation study

### Bull selection

An independent population of bulls was used to validate variant calls from whole-exome sequencing in bulls divergent for fertility. Fertility phenotypes of bulls from ICBF fertility records were used, as previously described. However, updated fertility data for this dataset from years 2013, 2014 and 2015 were available, which were not used for initial sample selection for TS and WES. In total, phenotypic information was available for 1,414 bulls over the three years. Of these, 123 bulls from the three breeds as used in WES (69 Holstein-Freisian (HO), 29 Limousin (LM) and 22 Belgian blue (BB) all with >100 insemination records per bull were selected for the validation analysis (lower insemination threshold was due to the shorter time scale over which recent data was collected).

### Assay design

Agena Bioscience MassARRAY^®^ System was used for assay design and SNP validation. SNP genotyping on the MassARRAY System combines the multiplexed primer extension chemistry of the iPLEX® assay with sensitive matrix-assisted laser desorption/ionization time-of-flight (MALDI-TOF)^44^. In total, 58 SNPs were targeted for validation in 4 multiplex reactions (29-, 18-, 8-, and 3-plex). DNA of each selected bull was obtained, and (10 ng) was used for each multiplex reaction. gDNA concentrations were estimated using a Nanodrop ND-1000 spectrophotometer. gDNA was dried down overnight in a PCR-free environment and shipped to Agena Bioscience GmbH, Germany.

### Validation data analysis

Of 58 SNPs targeted for validation in the MassARRAY assay design suite, 42 variants and 123 cattle passed filters and QC. SNPs which had a call rate <80%, or minor allele frequency (MAF < 0.04) were filtered out of the dataset. Statistical analysis of association was performed using PLINK v1.90b3l. Association analysis was performed with phenotypes as a quantitative trait using the Wald test statistic. A pedigree file was used to store animal pedigree and phenotypic data and a map file was used to store genetic marker data. This test statistic compares asymptotic allele frequencies between high-fertility and low-fertility (cases and controls).

### Preparation of oviductal explants

Reproductive tracts from non-pregnant crossbred heifers of beef breeds, were collected at a commercial abattoir immediately post-mortem and transported to the laboratory within 1 h in phosphate buffered saline (PBS) supplemented with Gentamicin sulphate (0.25 mg/ml, Sigma Aldrich, Wicklow, Ireland) at 4 °C. Reproductive tracts from heifers at various stages of the oestrous cycle were used, as stage has been shown to have no effect on sperm binding *in vitro* ^45,46^. At the laboratory, oviducts were trimmed free of connective tissue, and ligated using single use sterile umbilical cord clamps (Sutherland Health Group Ltd, Berkshire, UK). Oviducts were then washed twice with PBS following which the isthmic section of the oviduct was isolated. The epithelial cells were extruded in sheets by squeezing the oviduct with a sterile glass slide, fragmented by pipetting, centrifuged for 1 min (200 *g*), transferred to M199 culture media supplemented with fetal calf serum (10%) and gentamicin sulphate (2.5 mg/ml, Sigma Aldrich), and incubated for 1 h at 37 °C in 5% CO_2_ to form everted vesicles with apical ciliated surfaces oriented outwards^47^. Explants were used for binding assessments within 5 h of slaughter. Three reproductive tracts were flushed on each day and explants from each tract were processed separately.

### Sperm binding assay

M199 medium (5 mL) was added to explants from each tract and centrifuged at 200 *g* for 5 min. Post-centrifugation, the supernatant was removed and explants (20 µL) from each reproductive tract were added to sperm aliquots (140 uL) from high fertility bulls with (H + ive; n = 4 bulls) and without (H-ive; n = 4 bulls) the β-defensin haplotype as well low fertility bulls without the β-defensin haplotype (L-ive; n = 4 bulls). Sperm were pre-stained for 30 min with 1% (w/v) Hoechst 33342 at 37 °C prior to addition to the explants for enhanced binding visualisation. The final sperm concentration in the presence of the explants was 5 × 10^6^ per mL. After 30 min incubation at 37 °C in 5% CO_2,_ loosely bound sperm were removed from explants by gently pipetting through two droplets of M199 (75 µL) on a pre-warmed 24 well culture plate (37 °C). A droplet of each treatment (10 μL) was placed on a slide, a coverslip added and viewed using a microscope at 400X fitted with a heated stage at 37 °C (BX60; Olympus, Centre Valley, PA, USA) under half-light and half-fluorescence. The number of sperm bound was recorded and relative surface area of each explant was determined using a micrometre. Ten explants of each treatment were assessed at random for sperm binding density, calculated by determining the number of sperm bound per 0.1 mm^2^ of explant surface. The evaluator was blinded to treatment for all sperm binding assessments. Three reproductive tracts were used per replicate (replicate = day) and three semen straws per bull were assessed giving a total of twelve straws assessed per haplotype.

## Electronic supplementary material

Supplementary Info 1

Supplementary Dataset 1
