# Supplementary material for: A dual targeted β-defensin and exome sequencing approach to identify, validate and functionally characterise genes associated with bull fertility
Source: Sci Rep. 2017 Sep 25;7:12287. doi: 10.1038/s41598-017-12498-x (PMC5613009; doi:10.1038/s41598-017-12498-x)
Supplement: Supplementary file 1 — Supplementary Info 1 [file 41598_2017_12498_MOESM1_ESM.pdf]

A dual targeted β-defensin and exome sequencing approach to identify, validate and functionally characterise genes associated with bull fertility

Ronan Whiston[1¥], Emma K. Finlay[1¥], Matthew S. McCabe[1], Paul Cormican[1], Paul Flynn[2], Andrew Cromie[3], Peter J. Hansen[4], Alan Lyons[5], Sean Fair[5], Patrick Lonergan[6], Cliona O' Farrelly[7] and Kieran G. Meade[1*]

Supplementary tables

Table S1: Summary statistics for β-defensin sequencing

Table S2: Significantly associated SNPs from TS

Table S3: Summary statistics for WES

Table S4: Significantly associated SNPs from WES

Table S5: Gene-ontology analysis of SNPs divergent between fertility groups
